# “Ain’t It a Ripping Night”: Alcoholism and the Legacies of Empire in Salman Rushdie’s *Midnight’s Children*.

**DOI:** 10.1080/0013838X.2018.1436286

**Published:** 2018-04-06

**Authors:** Sam Goodman

**Affiliations:** Journalism, English and Communication, Bournemouth University, Bournemouth, UK

## Abstract

In the era of decolonisation that followed the Second World War, various authors sought to engage with India and the Empire’s past anew throughout their novels, identifying medicine and illness as key parts of Imperial authority and colonial experience. Salman Rushdie’s approach to the Raj in *Midnight’s Children* (1981) focused on the broad sweep of colonial life, juxtaposing the political and the personal. This article argues that Rushdie explores the history of colonial India by employing alcohol and alcoholism as lenses through which to explore the cultural, political and medical legacies of Empire. Through analysis of *Midnight’s Children* as well as a range of medical sources related to alcohol and inebriation, it will illustrate how drinking is central to Rushdie’s approach to secular and religious identities in newly independent India, as well as a means of satirising and undermining the supposed benefit that Empire presented to India and Indians.

Our Bombay: it looks like a hand but it’s really a mouth, always open.^1^

Salman Rushdie’s *Midnight’s Children* is as much an assault on the senses as it is a novel. Described by Abdulrazak Gurnah as a “grand” book both in ambition and in the scope of its subject, *Midnight’s Children* takes in nearly sixty years of Indian life and death on an epic scale, but crucially, on a human one too.^2^ Its skill lies in the juxtaposition of the personal journey of development, arrested or otherwise, of its protagonist, Saleem Sinai, with that of the newly independent India, both with their triumphs, both with their growing pains. As such, the book is an expression of a postcolonial literary consciousness, namely Rushdie’s own, as well as a representation of one, in the form of Saleem’s narrative.

In expressing such a perspective, Rushdie secured his place as one of the defining novelists of his generation, part of but also transcendent of a loose group of contemporary British and Indian writers including J. G. Farrell, V. S. Naipaul, Anita Desai, Ruth Prawer Jhabvala and others, who all sought to engage with the aftermath of British colonialism in their writing. Rushdie’s work in particular became a prominent dissenting voice in an era when British attitudes to the colonial Empire in India were overtly nostalgic in literature, television and cinema alike. As Andrew Teverson notes, Rushdie would seek to distance himself from what he called the “Raj Revival”, “the fantasy that the British Empire represented something ‘noble’ or ‘great’ about Britain; that it was, in spite of all its flaws and meannesses and bigotries, fundamentally glamorous”.^3^ Instead, novels like *Midnight’s Children* would provide a critical antidote to such revisionism, stripping away the veneer of opulence that glossed much of British colonial history.

Consequently, *Midnight’s Children* has long been considered a landmark text in the development of postcolonial literature and the Anglo-Indian response to Empire, receiving extensive and justifiable attention as a result. Indeed, the novel has been the subject of extensive literary criticism in general, and has been assessed in relation to a wide variety of topics.^4^ It is of course understandable that critical approaches to Rushdie’s fiction have focused largely on the postcolonial and political aspects of his work, especially when it comes to understanding the relationship between the past and present in novels such as *Midnight’s Children* or the more sharply satirical *Shame* (1983). Such approaches were driven by their historical and literary context, and remain instructive to subsequent engagement with the novel and interpretations of its multifaceted critique of Empire.^5^

However, it must be acknowledged that this scholarly focus has not, to date, included sustained analysis of the significant recurrence and indeed narrative predominance of medicine and health within *Midnight’s Children*, nor has the critical field addressed the utility of medicine more broadly as a means through which to read Anglo-Indian fiction produced after the end of the British Empire, or, further still, that of European colonialism.^6^ Exploring the presence of medicine and medical themes within Rushdie’s fiction, and their proliferation within the genres of post-Imperial and postcolonial literature, adds much to the existing critical discourses of race, power and identity that have so far been applied to Anglo-Indian fiction, and augments understanding of the experience of British India and the history and legacy of Empire.

As this article will illustrate, the history of British India reveals how health and medicine were defining preoccupations of Anglo-Indian society that have, in turn, influenced the representation of India within fiction. The British experience of India and its representation in print cultures both factual and fictional has always involved medicine and health. Ill health whilst in India is near universal in memoirs, journals, travelogues and diaries, with numerous medical texts, pamphlets and guides available on either how to avoid sickness or how to remedy it.^7^ Drawing on such sources, E. M. Collingham argues that the experience of India was intensely physical, marked by the centrality but also the transience of the body, with ill health and death central to Anglo-Indian life.^8^ Beyond the repeated instances of personal encounters with illness though, medicine and health can also be read in a colonial context as a principal means of control, social regulation and power; consider, for example, the authority vested in the Indian Medical Service and Indian Civil Service, whose responsibility for public health would enable them to restrict movement, prohibit religious or civic gatherings, or introduce other regulatory measures if they believed them conducive to public health, repeatedly demonstrating how the body was subject to British imperial power.^9^ Similarly, the tension between Indian and European methods of medicine and treatment are equally as illustrative of how medicine worked in a professional and also class-based context, with European doctors deemed of greater skill and social standing than their Indian counterparts, even if the native doctor had been trained in a European school of medicine.

*Midnight’s Children* contains multiple instances of illness, sickness and medical themes throughout its pages, from the recurrence of doctors, disabilities and the importance of birth, to the struggle with addiction. The fact that Rushdie, in addition to his other means, chooses to interrogate the legacy of Empire through a medical lens further places him in connection with those contemporary writers such as Farrell and Jhabvala who did the same, and medicine and medical humanities offer the opportunity to read Rushdie’s work, and that of his contemporaries, anew.^10^ To engage with medicine and health in a literary context, as Rushdie does and as medical humanities allows, is to continue to explore the history and social make-up of Britain’s colonial Empire, as well as the relationship between coloniser and colonised, from an alternative, yet no less significant, perspective.

To address this critical absence within approaches to Rushdie’s work, this article will draw attention to some of the medical themes at play within *Midnight’s Children*. It will focus on the use and abuse of alcohol within the narrative, and how this acts as a key metaphor through which to read the transition from a colonial to an independent India and the difficulties of partition, as well as the legacies of British social, legal and medical habits on the Indian body. Through close analysis of the published text of *Midnight’s Children* as well as Rushdie’s original manuscripts, and in conjunction with a range of medical sources related to alcohol and inebriation, the article will argue that Rushdie’s decision to employ alcohol and alcoholism so prominently is a means through which to undermine the general contemporary belief in the widespread benefits that British colonialism brought to India. Instead, the novel reveals the British presence as having had a debilitating effect on the physical, psychological and social health of newly independent Indian individuals and the wider nation alike. However, this analysis will suggest that Rushdie’s critique becomes double-edged. Rushdie not only portrays such legacies of British rule as destructive, but he also seeks to criticise the illusory ideals of the “New” India itself, drawing parallels between the fictional self-images both states indulge in. The British may have introduced Rushdie’s Indian drinkers to alcohol, but they remain enthusiastically complicit in the habit despite the British departure.

## Empire of the Setting Sun

Alongside the complexity of *Midnight’s Children*’s main narrative there are a host of significant and propitious subplots that contribute to, develop and intersect with Saleem Sinai’s story, as well as Indian history itself.^11^ One example is the handful of chapters that concern the circumstances of Saleem’s family’s move to Bombay and Saleem’s birth. Ahmed Sinai, Saleem’s father, and his wife are told by a friend how property is going cheap since the British are leaving in droves in advance of the declaration of independence, at this point only seventy days away. Ahmed meets and enters into a bargain to purchase a house on the estate of William Methwold, a British resident and descendent of the William Methwold of the East India Company, who lobbied for the acquisition of Bombay as a British territorial possession in the seventeenth century. The modern Methwold sells the four identical villas that comprise his estate on the condition that they come with all the contents, and that the new owners do not remove or alter any of the contents until the official declaration of Independence.^12^ Along with “talking budgies … moth-eaten dresses … used brassieres” and “half-empty jars of Bovril” come “cocktail cabinets full of good whisky”, which propel Ahmed on his journey towards the alcoholism that defines his character for the remainder of the novel.^13^

Alcohol, and Ahmed Sinai’s addiction to it, is one of the most prominent medicalised themes of the novel, revealing of Anglo-Indian relations, colonial history and culture.^14^ Returning to Collingham’s work on the embodied experience of health and medicine, her primary investigation is into what she calls the “Indianization” of the British or European body, mainly through a focus on “bodily practices” such as eating, dressing and grooming, but also by examining embodied difference by setting the body within urban and domestic space.^15^ However, whilst Collingham pays close attention to consumption in the form of food, she largely neglects the significance, and omnipresence, of alcohol in colonial society, presumably because it was a substance that Europeans brought with them and were familiar with, as opposed to something unusual or exotic encountered newly in India. However, alcohol is nonetheless important within the physical experience of India that Collingham identifies as it not only played a vital role in the perceived maintenance of health, but also, crucially, because it is another site of blurred racial and national identity, albeit one that throws her thesis in reverse; alcohol consumption, in *Midnight’s Children* and in colonial India, is a way of Anglicising the Indian body, and keeping the British body safely European, through the very same kinds of “bodily practices” that Collingham describes.

Like health and medicine, alcohol is central to depictions of Anglo-Indian experience, and is a recurrent trope of the same diaries, memoirs and journals that Collingham draws upon in her examination of the Indianised body. Alcohol played a role at all levels of society, consumed by all classes from native Indians and private soldiers (issued as part of rations until 1889, and then again during the First World War) through to colonial governors at vice-regal dinners, and variously helped grease the wheels of commerce, eased the burden of colonial service and acted as cause and remedy for multiple illnesses and ailments. In medical practice, alcohol was used to treat and prevent a number of conditions, and the choice of beverage would depend on the sickness. Beer, for instance, was thought to be nourishing as a result of its malt base and because it was made from the same ingredients as bread—this meant that it was often administered to those patients weakened by illness in a bid to restore their strength: a lay recommendation that lasted well into the twentieth century.^16^ Elsewhere, brandy was used to combat cholera up until the advent of bacteriology in the 1880s, and wine was often consumed in cases of fever.^17^ Drink in colonial India thus stood at a curious intersection of the social and the medical. However, there were prevalent fears of dissipation and social disorder associated with alcohol consumption, as well as a concern for the effect on health that regular excessive drinking engaged in by soldiers cost the East India Company and HM’s Forces, and the archives of the India Office contain numerous records of cashiering and invalidity because of intemperance and alcohol consumption.^18^

Alcohol and medicine are two distinct but connected means of gaining insight into British colonial Indian society, and its popular representation. In fiction, both from the height of the Raj and after 1947, drink often comes with a stern moral warning, or consequence, especially in the nineteenth century, and notably in the works of authors like Rudyard Kipling.^19^ This moral dimension reflected the medical history of alcoholism. Though historians such as Roy Porter have argued that knowledge of “drinking-man’s disease” and identification of its bodily markers existed as early as the eighteenth century, it was not until the mid-nineteenth century that studies by Magnus Huss gave alcoholism its name, and the condition (more often as “inebriety”) began to be considered and treated in a more clinical sense.^20^ Even so, despite this gradual pathology, addiction to alcohol was still generally perceived as a moral deficiency (a perception strengthened greatly by the Temperance campaigns of the nineteenth century) and thus as much a spiritual issue as a medical one.^21^ As Mariana Valverde argues, though the term “alcoholism” was in more widespread circulation in a medical and public context from the 1920s, it would not be until the mid-twentieth century and the development of Elvin Morton Jellinek’s Disease Concept of Alcoholism that the condition would become accepted into the Diagnostic and Statistical Manual of Mental Disorders and supported by research at the World Health Organization after 1950.^22^

Despite its recurrent presence, alcohol has, like medicine, been sorely under-explored in literary studies of Anglo-Indian fiction. Thomas B. Gilmore offers a potential explanation for this neglect in his book, *Equivocal Spirits*. Gilmore states that drink studies in literature are often, at best, peripheral or solely biographical, citing work on Ernest Hemingway, Charles Bukowski and Jack Kerouac, amongst others. Echoing cultural critic Frank Lettrichia, he goes on to argue that alcohol has been neglected because literary criticism has narrowed what is considered an acceptable topic for analysis.^23^ In considering Gilmore, the assessment of “bodily practices” that historians such as Collingham identify and the kinds of sources that they use to support their analyses, we can begin to see how medical humanities becomes an advantageous means of engaging equally with the medical evidence they present and the literary mode through which it is transmitted. Collingham and other historians of colonial India, such as David Arnold and Mark Harrison, employ diaries, journals, memoirs, handbooks and guides throughout their work as a means of exploring the history of the British Raj. However, it is as important to consider the culture of publication that enabled the printing of these sources and their status as narratives, as it is the sources themselves or their veracity.

What becomes clear is that sources such as these are not exclusively historical, and instead bear as much relation to literary works as they do factual documents. Like fictional works, these texts were prepared for a receptive audience, and as such were lucrative pursuits, especially if they were connected to any of the major wars or battles, such as the Indian Rebellion of 1857.^24^ They often follow an identifiable formula, told in first person, adopting a particular structure and style of writing and covering similar topics. This notion of writing both to and for an audience, the telling of a tale comprised of outlandish but also quotidian details, is what links these sources with a novel like *Midnight’s Children*. The same process of narrative transmission, a direct address (albeit, ostensibly to Padma within the novel’s fictional world) recounting a story of personal experience, results in both kinds of text covering the same topics, including health, medicine, eating and drinking in abundance. This suggests that the importance of medical humanities, and interdisciplinarity, here is in a dual and reciprocal opening up of source material; historical texts can be read as literary in their construction, and literary texts become representative of history or adopt a tangible historical consciousness, as Rushdie’s does with postcolonial attitudes to Empire and the past, offering new, interdisciplinary insight into the representation of colonial experience.

The same is true with *Midnight’s Children*, but Rushdie goes further and uses alcohol as a means of interrogating not only British history and experience, but also the power of British cultural hegemony during and after colonialism, and the state of Indian selfhood in the newly independent nation. Rushdie’s portrayal of Ahmed’s condition corresponds to a slightly simplified version of Jellinek’s concept of alcoholism, developed and refined between the 1940s and the publication of his book *The Disease Concept of Alcoholism* (1960). Initially categorising alcoholism into four stages, beginning with a pre-alcoholic stage through to full dependency, Jellinek’s later work would expand this into five stages (Alpha to Epsilon alcoholism), only some of which he considered pathologically to constitute “disease”.^25^ Rushdie depicts three identifiable stages in Ahmed Sinai’s decline into alcoholism in *Midnight’s Children*: a pre-alcoholic stage of intoxication, a Beta phase of inebriation through daily consumption which leads to social and physical problems, and finally a Delta/Gamma addiction involving inability to abstain and a loss of control. However, despite the correspondences between Rushdie’s portrayal and these clinical stages, the novel also reflects Gilmore’s assertion that alcoholism must be conceived of not only in medicalised terms but as a “total illness, with far-reaching effects that are not only physical but also psychological and spiritual”.^26^ Rushdie’s concern, expressed through alcohol, would be both for the body and the soul of the new Indian subject.

## Stage One: Pre-Alcoholism

The first of these stages, intoxication, is prompted by social interaction at the Methwold estate. In what he believes is a bid to secure the purchase of the estate, but, as the reader discerns, is as much to do with Methwold’s manipulation of him and delight in allegorical game-playing, Ahmed begins to spend more time with Methwold, who introduces him to the “cocktail hour”, something we are told has “never varied in twenty years”.^27^ Drawing on Gilmore’s remarks regarding the “acceptability” of certain subjects for literary study, if alcohol needs to be legitimated as a means of critique then it can be done so by considering it, and this episode, in relation to Homi Bhabha’s formulation of mimicry that he outlines in *The Location of Culture* (1994), a book that ties itself to *Midnight’s Children* from its own opening sentence. Bhabha argues that “mimicry emerges as one of the most elusive and effective strategies of colonial power and knowledge”, representing an “ironic compromise” between the demand for identity and the counter pressure of historical change.^28^ Mimicry of the coloniser offers, in Bhabha’s view, the pretence of visibility and identity, at the cost of authenticity to the colonised subject; they are rendered visible, but sacrifice a sense of fixed selfhood in the process. For Ahmed, alcohol becomes a means of enabling his visibility in the eyes of the coloniser (Methwold), but serves to render him, in Bhabha’s words, virtual, “almost the same *but not quite*”.^29^

There are two forms of mimicry going on in Rushdie’s text at this point: the cordial, homosocial surface mimicry of how Ahmed mirrors Methwold’s behaviour, but also that of a much more deeply ingrained mimicry within Anglo-Indian, or Anglicised-Indian class structures that his turn towards the bottle reflects. In relation to the former, Rushdie writes that “in the presence of the Englishman” Ahmed’s voice had changed; “it had become a hideous mockery of an Oxford drawl”, and he begins to invent stories of apocryphal Mughal ancestry, along with that of a family curse that would come to fruition, remaking his past (as Rushdie would later note of the colonised subject in *Imaginary Homelands*) to suit his present purpose.^30^ Mimicry thus becomes the realisation of power—the Estate, we are told, alters its inhabitants in both action and identity:

every evening at six sharp they all came out into their gardens or onto their verandahs because that was the cocktail hour, and though they were all Hindus or Muslims and Parsees and forbidden the juice of the fermented grape or any other hard stuff, they drank it anyway, and waved to one-another across their hedges, View-Halloo, they cried, Ain't it a ripping night.^31^

Such behaviour conforms, at least initially, to Bhabha’s notion that mimicry displaces power, here by showing up the ridiculousness of the colonial routine, the affected accents, habits and forced conviviality. However, at the same time, it embodies the idea of hegemony in the very public conformity of Ahmed, his family and their friends to expected and recognisable social roles, especially within what is still (until the seventy days are up) overtly British domestic space. They become not only intoxicated by drink, but by the heady lifestyle of the sahib, with the semi-public space of the verandah in which to demonstrate their ascendency. This spatiality, too, is significant—in studies of space and place, the domestic sphere is considered the “first space”, constitutive of individual being, of the true self away from the second space of the workplace.^32^ For Ahmed’s alcoholism to begin here demonstrates the extent to which the colonial project worked through domestic colonisation, as well as territorial. Alcohol reinforces this process, acting as a catalyst in breaking down the rules, religious or caste-based, by which they live and through which they determine their racial, social and political identities. Ahmed and the other residents are still socially and spatially othered, but their sense of Indianness is further eroded through drink, resulting in the mutability of the self. This mutability extends through time and space, and the negotiation of selfhood occurs in what is itself a transitional site between the public and private, outside and inside, and, at 6pm, the moment at which day fades into night. As such, their sundowners become a toast to Empire at the threshold of Imperial twilight, as the sun that never set finally goes down on colonial British India. Such resonances mean the episode also echoes the divisions in Indian identity that are exacerbated by partition. Beyond the barriers of the hedges denoting the separation between the families and their religions, the “ripping night” becomes an allusion to 15 August that entwines the personal and political in its reference to not only the tearing of the existing social and political fabric that occurred with Independence and partition, but also to the torn sheet that was responsible for the Sinai family dynasty. Ahmed and the other residents are figuratively torn between their origins and their newfound distinction, their Indianness and their Anglicisation.

The notion of domestic colonisation extends to the other layer of mimicry taking place in this section of the novel. Rushdie’s text speaks to the legacy of “cultured” British society on India in the trace of the so-called “Macaulay Indian” that Methwold and his actions attempt to embody in Ahmed. In *Self, Nation, Text in Salman Rushdie’s Midnight’s Children,* Neil Ten Kortenaar details the influence of Thomas Babington Macaulay’s infamous “Minute on Indian Education” from 1835, and his attempt to create “a class of persons Indian in blood and colour, but English in tastes, in opinions, in morals and in intellect”.^33^ Kortenaar explores how the objects that clog the villas influence the behaviour of their occupants and whilst he acknowledges the importance of the cocktail hour, he overlooks the significance of drinking in relation to the mimicry he identifies elsewhere in the novel and inherent to Macaulayism in general. Methwold’s interactions with Ahmed are framed, perhaps a little more overtly in the manuscript version of the novel but present nonetheless in the final text, around a sense of instruction, and its long-lasting effects. Methwold (or “Westmead” as he is in the “first draught”) “would come round to teach my father to hate all full bottles of scotch and to dedicate himself to the emptying of these monsters which imprisoned the fluid object of his love”.^34^ Moreover, Methwold’s act of leaving the cocktail cabinets full of good whisky, Saleem states, “thus [introduced] my father to what was to be the greatest and truest love of his life, the onlie [sic] begetter of our ensuing family atmosphere”.^35^ Drinking becomes integral to Ahmed’s mimicry, and is a reflection of how vital drinking, and more specifically drinking whisky, was to denoting the Indian middle classes. Gina Hames states in *Alcohol in World History* (2012) that alcohol was both a mark of westernisation and a status symbol, noting that alcohol imports to India increased 900 per cent between 1875 and 1928, meeting the demands of a newly Anglicised Indian middle class, such as the Sinais.^36^

The mutability of the Indian in the face of British influence is acknowledged by Rushdie, who writes of how Methwold “presided over this blending of superior western civilisation with good honest native stock! A glorious age, he must have felt, was being given a glorious end”, in so much as his efforts leave behind a lasting legacy embodied in the habits impressed upon Ahmed.^37^ Note too the resonances of the linguistic choices Rushdie makes here—the action is one of “blending”, suggestive of hybridity, even of passing and “blending in”, but also of whisky terminology. A blend is substandard, inferior, yet at the same time the act of mixing makes its base ingredients more palatable. The kind of abasement that alcohol engenders within Ahmed does much to undermine the notions of resistance that Bhabha argues are inherent to mimicry. Alcohol erodes Indianness in a way that other forms of Anglicisation such as education or language do not. Education and language can be turned back against the oppressor, as indeed they were by the Indian nationalist movement from the early twentieth century onwards, whereas the tide of alcohol that begins to wash Ahmed’s identity away is irresistible. Of course, mimicry still works in two directions, and Methwold himself is held up for ridicule with his pronunciation of “Sabuckuck theek-thaak hai” (an Indianised saying meaning, ironically, “everything’s just fine”, but also evocative of the countdown to Independence, and the section’s title) and vanishes, never to be seen again; however, as the book progresses it emerges that the effects of his “little game”, like those of Mountbatten’s, have long-lasting implications.^38^

Rushdie’s decision to begin Ahmed’s decline into alcoholic dependency at the point of political and national emancipation illustrates his own complex relationship to Indian identity, and Indian history. On the surface, it appears as a straightforward criticism of the decadent and disempowering influence of the British; the fact that Methwold introduces Ahmed to the whisky that then precipitates his decline is a pointed reference to the negative influence of the British presence on Indian culture, seeing as it damages his personal health, his marital and professional relationships, induces him to break his religious vows, and dilutes his Indian identity. However, given the repeated statements on the importance of allegory throughout the novel, Ahmed himself, and the class and generation of Indians he represents and belongs to, are also the subject of Rushdie’s criticisms. Ahmed, like Nehru and Gandhi, strikes a bargain with the British that whilst seemingly empowering has destructive and unforeseen consequences. Just as Gurnah observes of Nehru, that his “ambition for post-independence India begins to disintegrate almost as soon as India is founded, in the partition violence and the language marches”, Ahmed’s decline and disintegration begins at exactly the same point.^39^ It is an example of how, like in the Anglo-Indian fictions of Scott, Farrell and Jhabvala, the relationship between the individual and the nation is so often read through the prism of medicine and health, supporting Jeffrey Meyers’ observation that the sickness of the hero is often analogous to the sickness of the state.^40^

## Stage Two: Alpha/Beta

The second stage of Ahmed Sinai’s alcoholism begins after the formal declaration of Indian independence. As Saleem explains:

cocktail cabinets had whetted his appetites, but it was my arrival that drove him to it … In those days, Bombay had been declared a dry state. The only way to get yourself a drink was to get yourself certified as an alcoholic; and so a new breed of doctors sprang up … [namely] Whisky Doctors.^41^

Saleem goes on to detail how Ahmed, and most of the “respectable” men of the neighbourhood would queue up each month at Dr Sharabi’s surgery and emerge with the “little pink chitties of alcoholism”.^42^ However, the ration soon proves inadequate for Ahmed’s needs and he begins to send his servants along to the doctor too, and the gardener, before resorting to paying for further supplies, with Rushdie writing that “the poor, having little else to peddle, sold their identities on little pieces of pink paper; and my father turned them into liquid and drank them down”.^43^ With his reliance on whisky becoming more acute, Ahmed further assumes the habits of the now departed Methwold, turning to philandering and making a series of ill-advised business decisions.

Again, the change wrought upon Ahmed in this section can be read initially through the productive context of mimicry. In introducing his concept of mimicry, Bhabha draws on the work of Jacques Lacan, beginning his chapter with an epigram that reads: “The effect of mimicry is camouflage … it is not a question of harmonizing with the background, but against a mottled background, of becoming mottled”.^44^ The linguistic and associative resonances with Rushdie’s description of Ahmed’s alcoholism are evident. The notion of his becoming “mottled” in his mimicry evokes not only the mottled complexion of the habitual drinker, but also the dimple bottles he draws from Methwold’s cabinets, with the confluence between the drinker and the drink another way in which he is “blurred at the edges” by his burgeoning dependency.^45^ Similarly, we are told of the “whisky doctor” “Dr Sharabi’s mottled-glass surgery door” itself suggestive of opacity, of hidden practices such as the black market trade that drives the selling of ration chits, and of secret drinking behind closed doors in the newly dry state of Bombay.^46^

Moreover, the sense of the mottled background Lacan refers to is reflected in the diffusion (or dilution) of Ahmed’s identity as a result of the methods he employs to obtain his alcohol ration. Rushdie writes of how Ahmed is known at

six different stores under six different names. He was a Hindu, a Muslim, a Sikh, a Jain and two Parsees for the sake of a drink. For whisky’s sake he became a teacher, an ice-cream salesman, a monk, a keeper-of-a-Tower-of-Silence and a swimming-bath-attendant as well as a property-owner-and-entrepreneur.^47^

In this stage, Ahmed’s drinking becomes a form of erasure, leading on from his mimicry, in which he begins to lose control, and loses his grip on his identity. Whereas in the first stage and during his interactions with Methwold, Ahmed alternated between public and private states: the “Macaulay Indian” with Methwold, and “Janum” with his wife Amina—here his identity becomes less stable, becoming refracted through the facets of the whisky bottle and into these various illusory roles. Ahmed’s mimicry then becomes paradoxical. He is credible enough to pass as these different individuals, to “blend in” in these different contexts, but in so doing becomes less distinct at the same time—as Rushdie writes: “Ahmed Sinai blurred the edges of himself by drinking”.^48^ He becomes all and none of those other identities at once, never truly any of them, and the act of trying to become other renders his own sense of stable self diluted, watered down. Yet, there is still stability present in his clinical identity; he is officially (if ironically not in the clinical definition, yet) “an alcoholic”, and his identity as such has been diagnosed, certified and negotiated by a combination, or another blending, or even a mixture, of Bombay’s medical, legal and social practices. There is a contradictory play of control and power going on here, in so much as Ahmed is able both to replicate other identities but also become the subject of replication himself.

In his description of Ahmed’s “sickness”, and of his multiplicity, Rushdie again draws comparisons between the state of the individual and the state of India. Ahmed takes on these different guises, and in doing so becomes all India, suggesting how the desire for alcohol, and indeed addiction and its medical definitions, all act as unifiers, cutting across boundaries and divisions of religion, occupation and caste. However, again class and social rank complicate Rushdie’s depiction of Indian unity. Ahmed, as a middle-class entrepreneur and “Macaulay Indian” of course has the freedom to do this, and his economic status enables his drinking through the exploitation of the poor—ironically, his adoption of “low class” identities is at odds with the middle-class habit of whisky drinking. Such exploitation of the lower classes again puts drinking in the realms of bankruptcy—both in the figurative moral sense that Ahmed courts through exploiting those below him in social standing and through his philandering, but also in the literal sense of where his drinking takes him; by the end of this section, he has been ostracised from the business community, his assets frozen, with only the option of further erasure, to declare himself bankrupt, as a means of escaping his predicament.

Beyond the secular, social aspect of Ahmed’s alcoholism, this section of the novel is particularly interesting for the spiritual dimension to drinking that it introduces. The changes that Rushdie makes to this section in the manuscript and final versions of the novel at first appear superficial, but when read in the context of drinking are shown to have significant symbolic depth. In the “First Draught”, Rushdie writes of Ahmed’s dependency with reference simply to “whisky”. However, in a successive version he introduces the term “djinn”—an Arabic word that has a range of interpretations including genie, demon or, more suggestively, a malevolent or “bad spirit”.^49^ Such a choice of diction is redolent of multiple metaphorical associations. To free a genie from a bottle often brings unintended consequences as well as initial riches; the term evokes ideas of the “demon drink”; and it is also homophonous with that famed colonial beverage and quasi-medical indulgence, gin and tonic. Following on from Methwold’s playfulness around independence, ironically undercut through tying Indians to an English way of life, the novel preserves its prevailing sense of allegory in relation to the djinns. Saleem recounts how Ahmed tells him a story of a fisherman who found a djinn in a bottle washed up on the shore, warning him “let them out of the bottle and they’ll eat you up”, whilst all the while clutching a “green bottle with a white label”. In telling his classmates of his father’s actions the next day, Saleem is met with ridicule: “My father fights with djinns; he beats them … and it was true. Ahmed Sinai … began, soon after my birth, a life-long struggle with djinn bottles. But I was mistaken about one thing; he didn’t win”.^50^

This section of the novel can thus be read, in light of Gilmore, as a point of spiritual and political crisis for the new Indian state; a battle for the “soul” or “spirit” of the Indian subject embodied in Ahmed, just as much as it is in Saleem as a midnight’s child. Medicine too, is transformed, and the “whisky doctors” of the first draught become “djinn-doctors”, responsible for the soul as well as the pathologised body. Through Ahmed, his drinking and the wider context of state-enabled alcoholism, Rushdie criticises the notion that the “birth” of a new nation does away with the compromise, and the weakness of old. The “new” India, for all its patriotic zeal and moralising, is shown by Rushdie to be as complicit in the behaviour it formerly condemned. The new legislation that turned Bombay into a dry state, itself a rejection of Macaulayism and part of a constitutional promise to prohibit intoxicating drink and drugs (except “for medicinal purposes”) is shown to be impossible to enact, and undermined by those who supposedly desire its implementation.^51^ Alcohol becomes another means of criticising the gulf between the impossible and illusory ideals of the new India and its reality, just as Rushdie does elsewhere in the novel with nationalism and propaganda through Jamila Singer’s role in Pakistan and later government, and the clearance of the slums in which hundreds die. Moreover, it is not the British that push Ahmed into alcoholism, but rather the actions of his fellow Indians. As a Muslim and a vulnerable minority, Ahmed faces financial persecution by Bombay’s Hindu businessmen, including having his assets frozen and pushing the family into considerable financial difficulty, which exacerbates his drink dependency. Rushdie’s point again is that the idea of unified India as one state, as declared by the nationalists, is fallacious.

## Stage Three: Gamma/Delta

The final stage of Ahmed Sinai’s alcoholism is clinical addiction. Progressing beyond the pre-alcoholism of the earlier sections, Ahmed’s behaviour begins to advance through the various phases of Jellinek’s scale, shifting from the Alpha and Beta categories, where drinking occurs first as a response to social or emotional problems and then involves heavy drinking on a daily basis, and then into the more advanced Gamma and Delta phases. In Ahmed’s case, many of these symptoms begin to appear in the years following the seizure of his assets, particularly in the form of his increasingly erratic behaviour in the home and at work. However, again corresponding to Jellinek’s phases, Saleem recounts how, at this stage, although “djinn-sodden” Ahmed’s drink problem is manageable enough for him to still function.^52^ In fact, his drinking gives him the confidence to sell what property he has left and use the money to play the stock market to some success, with Saleem noting how it was “a feat made more remarkable by Ahmed Sinai’s worsening drinking habits”.^53^

Unlike the first two states, the grip of Ahmed’s addiction tightens at a much faster pace in this section of the novel, and Rushdie writes that “his financial coups obscured his steady divorce from reality … under cover of his growing riches, his condition was getting steadily worse”, to the point where “by July, Ahmed Sinai had entered an almost permanent state of intoxication”.^54^ This permanent state puts Ahmed on the path towards the more advanced stages of the disease concept; those phases which Jellinek considered constitutive of full-blown addiction. The earlier stages, in his opinion, constituted a state of physical dependency but not, crucially, a loss of control. However, despite the nuances of Jellinek’s diagnoses, Ahmed reaches a point where the dilution of his identity and the indistinct self of the previous sections are replaced by an intensity of emotion, and he experiences extremes of despair, despondency, malice, anger, romance and manic activity, alongside his financial success.^55^ Gilmore’s assertion that alcoholism is “total illness, with far-reaching effects that are not only physical but also psychological and spiritual” can again be observed in Ahmed’s swift decline. He begins to believe in the family curse he invented as part of his mimicry, and at one point is found trying to transfer his misfortune to the family dog: “This was that same fictional curse which he’d dreamed up to impress William Methwold, but now in the liquiescent chambers of his mind, the djinns persuaded him that it was no fiction”.^56^ Whereas before the fluidity and confluence between drink and drinker extended to Ahmed’s legal and social identity, alcohol now begins to unmoor his mind and his psychological stability.

Such developments signify progression into the Gamma phase of the Jellinek scale. In the Gamma phase, according to Valverde, the alcoholic begins to develop tissue tolerance and dependency, and suffer from a loss of control.^57^ The most significant shift in this section is how the effects of Ahmed's drinking are portrayed. Rather than a change to his mannerisms, company, identity or behaviour, in the Gamma phase the alteration is writ large in bodily signs of decline and damage. For instance, Saleem notes for the first time that “Ahmed Sinai’s face [was now] ravaged by whisky” and that his “battles with djinns” had left him dusty, unwashed and with blood-rimmed eyes.^58^ Ahmed’s self-inflicted “mottling” shifts from the social to the physical.

Ironically enough, this shift in Ahmed’s status towards full-blown disease-concept alcoholism occurs after the system of medicalised alcohol rationing has ended and the certification of alcoholics is no longer necessary. Prohibition in Bombay was enacted between 1948 and 1950, and then again after 1958, being repealed each time to combat the increase in criminality that it produced.^59^ This shifting political and legal landscape renders Ahmed an officially certified alcoholic when clinically he is not, and then leaves him clinically an alcoholic when certification no longer matters. Despite the ironic tone to this diagnosis, Rushdie appears to be making a significant point regarding medicine and its effects on the individual. If we are to read Ahmed in terms of the allegory with which we encounter him throughout the novel, then his condition is a reflection of how medicine can be manipulated by those with influence or power, but has severe shortcomings when it comes to improving the quality of provision and treatment when it is actually required.

The final part of Ahmed’s role in the novel involves a further legacy of alcohol. Saleem receives a telegram whilst in Pakistan urging him to return to Bombay as soon as possible because his father has been diagnosed with a “heartboot”, something later explained as a swollen lower left ventricle, causing the heart to look quite literally like a boot.^60^ This condition, also known as cardiomegaly, has multiple causes, one of which is long-term alcohol abuse.^61^ The shock of the “heartboot” sends Ahmed into recovery, and, tended by his wife, Saleem explains how,

not only did Ahmed Sinai make a recovery so complete as to astound Breach Candy’s European doctors, but also an altogether wonderful change occurred … under Amina’s care he returned not to the self which had practised curses and wrestled djinns, but to the self he might always have been, filled with contrition and forgiveness and laughter and generosity and … love.^62^

However, despite this near miraculous recovery and the swing back from addiction to a comparative state of rejuvenation, Ahmed cannot escape the legacy of his past. Having re-established his relationship with his wife, he and Amina move, again with a sense of spatial metaphor, to another dry state, Pakistan, and begin a successful towelling business (another allusive means of becoming dry). Not long afterwards though, Ahmed suffers a debilitating stroke, again as a result of the long-term physical strain of his drinking, and is rendered near insensible as a consequence, before then being killed by bombing in the Indo-Pakistan war of 1965.

In considering Ahmed’s narrative arc, *Midnight’s Children* is again found to be both aware and concerned deeply with reflecting history, especially in terms of generic convention and the writing of embodied experience found in Anglo-Indian print cultures. For instance, Rushdie’s novel echoes the kinds of stern warnings when it comes to drink of the literature that precedes him. Though his approach does differ in that the moral dimension, familiar in Kipling and others mentioned earlier, is largely absent here; although Ahmed has many failings as a result of his drinking, Saleem, and by extension Rushdie, do not frame Ahmed’s addiction in terms of moral weakness, nor do they pass judgement on his character and his flaws. However, the consequences, ranging from the social, professional, familial and medical are made apparent repeatedly all the same. Ahmed’s story ends in tragedy on various levels, with so much of his life effectively lost to substance abuse, and then having his second chance also taken away by what is a medical, pathological hangover from his drinking.

Similarly, and in a link to the kinds of narratives employed by historians of medicine and bodily practices in India and colonial history, we can view Ahmed’s decline in *Midnight’s Children* in terms of the familiar memoir, diary and written record of Indian life. Ahmed’s relationship with alcohol can be seen to mimic and imitate the narrative structure of the kinds of sources employed by Collingham and other historians in their explorations of embodied experience of India. Ahmed’s decline begins with surprise and transformation, moving to a sense of acclimatisation, before eventual ruin and near dissolution which forces him to leave India to achieve some degree of rehabilitation. Echoing such a structure, and embodying it in a character whose unstable national identity fluctuates as a result of the force of history as well as his personal mimicry, Rushdie appears to apply the tropes of literary postmodernism to the genre of Anglo-Indian writing. The reuse of form, theme and imagery are recurrent tropes of the postmodern project, and conform to Rushdie’s satirical and ironical commentary on the British and Indian past, as well as the means of communicating any kind of personal and historical narrative, as demonstrated in Saleem’s retelling. Alcohol becomes integral to such an account; a central ingredient of the British romanticism of Empire, Rushdie uses it here to explore a more squalid side to the same story.

## Conclusion

In this article, I have illustrated how alcohol, and medicine, can be productive lenses through which to read Anglo-Indian and postcolonial fiction such as Rushdie’s *Midnight’s Children*. *Midnight’s Children* is a novel about history: personal and political. Indian history is so often about how medicine interlinks these two categories, from the grand sweep of nation building to the quotidian details of day-to-day life and death. For Rushdie, to exclude health, medicine and illness would be to miss the essential humanity of Indian history, something lacking in so many of the depictions of India and British colonialism in the Raj Revival. As in the work of his contemporaries such as Farrell, Scott and Jhabvala, by becoming illustrative of their bodily or psychological weaknesses medicine serves to make the characters within the novel that much more real; illness is human, and such failings are what bring characters to life.

Medicine and alcohol are vital ingredients of that history and that process too. *Midnight’s Children* seeks to represent the history of India under colonial rule and after, and to do that accurately or fully includes activities such as drinking. The novel abounds with the legacies of British rule; drinking is a means of suggesting that these legacies, whilst innocuous enough in small doses, are revealed to do great damage over time. Drinking is shown to be as ubiquitous in Rushdie’s fiction as it was in Indian society, and enables Rushdie to explore several considerations and complications around ideas of social, religious and national identities, all unified and divided in simultaneity in a newly independent India. Ahmed’s new found freedom on taking possession of the house in Bombay is both blessing and curse—offering the freedom of self-determination, and the freedom to self-destruct. In a similar fashion to what he does with Saleem and the other Midnight’s Childrens’ powers, Rushdie uses alcohol to allegorically suggest independence as one of the new India’s djinns—the end of Empire precipitates the release of a genie from a bottle that grows into a moral, physical and spiritual battle for the health and soul of a nation. Where the other legacies and indignities of British rule are gradually assimilated, alcohol and drinking proves exceedingly difficult to swallow.

## Footnotes

1 Rushdie, *Midnight’s Children*, 125–6.

2 Gurnah, ed., 91.

3 Teverson, 7.

4 For a variety of approaches to *Midnight’s Children* and Rushdie’s work in general, see Gurnah; Freigang; Goonetilleke; Eaglestone and McQuillan, eds; Cundy.

5 As a range of commentators have noted, the historical novel experienced a boom in popularity in the decades after the Second World War; whilst contextually driven by what Fredric Jameson called the “nostalgia industry”, the desire to engage with the past through narrative or literary means seems acutely British, and correlative to the decline of Empire. See Boccardi.

6 Such approaches that do exist are limited in scope. Alberto Fernández Carbajal mentions Jean M. Kane’s assessment of western and Ayurvedic medicine acting as a physical metaphor for the clash of nations and traditions within the novel, as well as Clare Barker’s work on the recurrence of disability. See Fernández Carbajal, 222.

7 These vary from specialist publications on health, such as Edmund C. P. Hull’s *The European in India*, to more general works which combined medical information with practical details of travelling and recommendations for tourists such as *The Imperial Guide to India, Including Kashmir, Burma and Ceylon*.

8 Collingham, 1.

9 Mark Harrison writes of the various attempts to improve standards of public health across British colonial India. Whilst those engaged in such practices believed themselves to be doing good, their methods often bred resentment amongst those whose lives they were intending to improve. See Jackson, ed., 285–302.

10 There are a number of examples of how medical approaches can illuminate new significances in familiar literature and augment well-established literary disciplines; for example, see Stiles.

11 It remains debatable as to whether a main narrative exists in the novel; despite the presence of a protagonist and the bildungsroman-esque nature of the book, its form and preoccupation with how the past intrudes on the present precludes a sense of linear narrative. However, Ralph Crane argues that such framing is a consistent trope of Anglo-Indian fiction, noting that novels by Paul Scott and Ruth Prawer Jhabvala, and Richard Attenborough’s *Ghandi* (1982) all begin in the present before going backwards within narrative time. See Crane, 88.

12 *Midnight’s Children* “First Draught” [sic], 103–6, MSS/1000, Box 15/10, Salman Rushdie Papers, Stuart A. Rose Manuscript, Archives, and Rare Book Library, Emory University (hereafter *Midnight’s Children* “First Draught”).

13 Rushdie, *Midnight’s Children*, 98; *Midnight’s Children* “First Draught,” 106,.

14 Alcohol is present throughout the novel, as well as in the sections under discussion here. Even the book’s arch-villain, Reginald Dyer, the man responsible for giving the order that led to the massacre of unarmed civilians at Amritsar in 1919, has an association with alcohol. Dyer was an Anglo-Indian, born in Muree in the Punjab (now Pakistan), where his father worked as a brewer; see Collection 233/2 Beer supply, Madras and lower Burma: questionable economy of accepting tender of Nilgiri & Muree Brewery Co. IOR/L/MIL/7/9936: 1884–6, India Office Archives, British Library.

15 Collingham, 2–3.

16 Gately, 78.

17 There are a wide range of lay journals and medical texts that suggest the use of alcoholic drinks either as general specifics, or as part of treatment for various illnesses; see Wade; Foster; Russell; Hooper; Fenwick.

18 See Military Department Special Collections, IOR/L/MIL/5/376, Coll 4a: 1810–13; Courts martial of British officers in India 1861–75, IOR/L/MIL/5/674, India Office Archives, British Library.

19 An example lies in the air of dissipation that surrounds Hummil, the British engineer stuck in a remote station in Rudyard Kipling’s *At the End of the Passage* (1891); Hummil’s drinking habits exacerbate his unhappiness, to the point where Spurstow, the station doctor, unloads his gun lest Hummil do himself harm.

20 Porter.

21 Valverde, 66. See also Herring *et al*., eds.

22 Valverde, 99, 111, 114.

23 Gilmore, 12. Rachel Black also notes the historical conflation of moral and medical discourse around alcohol addiction, but suggests gradual growing social understanding of the pathology of addiction has changed this perception; Black, 6.

24 See Klaver; Goodman, “Lady Amateurs and Gentleman Professionals.”

25 Valverde, 111–12.

26 Gilmore, 5.

27 Rushdie, *Midnight’s Children*, 95.

28 Bhabha, 122.

29 Ibid., 123.

30 Rushdie, *Imaginary Homelands*, 33.

31 *Midnight’s Children* “First Draught,” 106.

32 Martin, 93–107. Martin goes on to argue that the pub or alehouse is the “third place, and constitutive of social identity”.

33 Kortenaar, 168.

34 Rushdie, *Midnight’s Children* “First Draught”, 106. The alteration from “Westmead” in the “First Draught” to the more historically resonant “Methwold” in the final published version happens swiftly in Rushdie’s revisions. Presumably the increased closeness to historical fact suited the novel’s approach to historiographic metafiction and magical realism.

35 Ibid., 106.

36 Hames, 91. D. C. R. A. Goonetilleke notes that the transfer of power on Methwold’s departure passes to those Indians already predisposed toward the culture of the West, however, again the cross-section of religions and occupations of the group suggest that such a predisposition can exist in any Indian, and not just a particular group. See Goonetilleke, 26.

37 Rushdie, *Midnight’s Children* “First Draught,” 107. Sara Upstone draws on Bhabha to note that the colonised body is “paradoxically a site of attraction and abjection, a location ‘always simultaneously inscribed in both the economy of pleasure and desire and the economy of discourse, domination and power’”; indeed, in Methwold’s games, as well as his affair with Vanita, the colonised body becomes this site of inscribed domination and desire, tempered with the knowledge of impending loss of power. See Upstone, 150.

38 Methwold reappears in self-reflexive, postmodern fashion in *The Ground Beneath Her Feet* (1999). Chronologically earlier than *Midnight’s Children* and presumably in the same fictional universe, Methwold appears as a scholar of comparative mythology, for approximately ten pages (41–51). Though much changed from his portrayal in *Midnight’s Children*, he is still associated with alcohol, drinking whisky with Sir Darius Xerxes Cama, and the novel has Sir Darius succumb to drink and addiction like Ahmed Sinai does. The character is definitely Methwold, but a strangely less patrician, more intellectual character than before (though one whom also retains, apparently, his taste for Indian lovers).

39 Gurnah, 95.

40 Meyers, 4–5. See also, Goodman, “A Great Beneficial Disease.”

41 Rushdie, *Midnight’s Children*, 131–2.

42 Ibid., 132.

43 Ibid.

44 Bhabha, 121.

45 *Midnight’s Childreni,* TS: Box 16/10, 150, Salman Rushdie Papers, Stuart A. Rose Manuscript, Archives, and Rare Book Library, Emory University.

46 *Midnight’s Children*, TS: Box 17/3, 139, Salman Rushdie Papers, Stuart A. Rose Manuscript, Archives, and Rare Book Library, Emory University.

47 Rushdie, *Midnight’s Children*, 150.

48 Ibid., 132.

49 The Quran states that djinns are made from a “smokeless fire” much like that which alcohol gives off when burnt, as Ahmed does in the novel; Abdel Haleem, trans., 55, 14–15; Rushdie, *Midnight’s Children*, 131. In *On the Best Means of Preserving Health in India*, Surgeon General C. R. Francis states that the term “jin” shares no etymological root with alcohol, however, “its demoniacal character may render it deserving of such”; Francis, 3.

50 Rushdie, *Midnight’s Children*, 131.

51 Hames, 92.

52 Valverde, 111. Rushdie, *Midnight’s Children*, 203.

53 Rushdie, *Midnight’s Children*, 202.

54 Ibid., 203.

55 In the “First Draught”, Homi Catrack explains how he is not keen on liquids because they mean dilution whereas he believes in concentration and intensity, however, he is persuaded to drink neat whisky and finds it “a wonderfully intense experience”, *Midnight’s Children* “First Draught”, 106–7.

56 Rushdie, *Midnight’s Children*, 203.

57 Valverde, 112.

58 Rushdie, *Midnight’s Children*, 236, 283. Ahmed’s decline and alcoholism corresponds to colonial assessments of the native Indian capacity for drink; Francis states that the native physique is incapable of managing alcohol consumption, and such consumption results in “the worst passions of human nature being let loose”; Francis, 47–8.

59 There were earlier attempts at prohibition, including during British rule in Madras in 1937. See Phillips, 276; Sharma, Tripathi, and Pelto.

60 Rushdie, *Midnight’s Children*, 294.

61 See Baskin, 212.

62 Rushdie, *Midnight’s Children*, 297.
